# Improved detection of genus-specific *Alphavirus* using a generic TaqMan® assay

**DOI:** 10.1186/s12866-017-1080-9

**Published:** 2017-07-24

**Authors:** Claude Giry, Bénédicte Roquebert, Ghislaine Li-Pat-Yuen, Philippe Gasque, Marie-Christine Jaffar-Bandjee

**Affiliations:** 1 Centre National Arbovirus Associé, CHU de la Réunion-Site Nord, Saint-Denis, La Réunion France; 2 Laboratoire de microbiologie, CHU de la Réunion-Site Nord, Saint-Denis, La Réunion France; 3 Laboratoire d’immunologie clinique et expérimentale ZOI (LICE-OI), CHU de la Réunion-Site Nord, Saint-Denis, La Réunion France; 4 UMR PIMIT, Processus Infectieux en Milieu Insulaire Tropical, Plateforme Technologique CYROI, Sainte-Clotilde, La Réunion France

**Keywords:** *Alphavirus*, Molecular diagnosis, Virus emergence

## Abstract

**Background:**

Alphaviruses are arthropod borne RNA viruses of medical importance. Geographical expansion of mosquitoes of the *Aedes* genus in the past decades has been associated with major *Alphavirus*-associated outbreaks. Climate changes and intensification of air travels have favored vector expansion and virus dissemination in new territories leading to virus emergence not only in tropical areas but also in temperate regions. The detection of emergence is based upon surveillance networks with epidemiological and laboratory investigation.

**Method:**

A specific, sensitive and rapid screening test for genus-specific *Alphavirus* is critically required. To address this issue, we developed a new molecular assay targeting nsP4 gene and using a TaqMan® real time RT-PCR method for the specific detection of all major *Alphavirus* genus members.

**Results:**

This assay was tested for specificity using several *Alphavirus* species. We also tested successfully clinical sensitivity using patient’s samples collected during the *Chikungunya* outbreak of 2005–2006 in the Indian Ocean.

**Conclusions:**

This new pan-*Alphavirus* molecular diagnostic tool offers great potential for exclusion diagnosis and emergence detection given its broad specificity restricted to *Alphavirus* genus.

## Background

The genus *Alphavirus* belongs to the family *Togaviridae* and comprises 29 virus species. *Alphaviruses* have a positive single stranded RNA. All of them are arthropod-borne except salmon pancreatic disease virus (SPDV) and southern elephant seal virus (SESV). Transmission involves intermediate host from avian or mammal origins for virus replication and mosquitoes as vectors for spillover infection in humans. *Alphaviruses* form icosahedral structures of 70 nm in diameter that contain a 5′ capped- and 3′ polyadenylated genome. Virus genome is ranging from 10 to 12 kb in length and comprises 2 open reading frames (ORF) separated by an intergenic sequence. 5’ORF encodes a polyprotein comprising 4 non-structural proteins (nsP) 1 to 4 and is involved in a replicative complex. The subgenomic RNA encodes for instance 3 structural proteins, E1, E2 and Capsid that interact to form virion envelope [1]. *Alphaviruses* form a small subset among all reported arboviruses. In the international arbovirus catalogue maintained by the Centers for Disease Control and Prevention (CDC – Atlanta), 537 viruses are actually reported; 9 of them are known to be *Alphaviruses* of medical relevance, *Chikungunya* virus (CHIKV), *Ross river* virus (RRV), *O’nyong nyong* virus (ONNV), *Sindbis* virus (SINV), *Mayaro* virus (MAYV), *Barmah forest* virus (BFV), *Eastern equine encephalitis* virus (EEEV), *Western equine encephalitis* virus (WEEV) and *Venezuelan equine encephalitis* virus (VEEV) [2].

Large outbreaks of CHIKV disease were documented in Gabon (1999), Indian Ocean (2006), India and Southern Asia (2006, 2007) [3–7]. ONNV was restricted to Africa until an imported case was described in a German traveler who stayed in Kenya [8]. MAYV was isolated from a human case in Trinidad (1954) then in several parts of South America and recently in French Guiana [9]. BFV is endemic in Western Australia as well as RRV. RRV was responsible for a large outbreak in Australia and in Western Pacific Ocean Islands; SINV related disease was widely distributed and associated to outbreaks in Africa, North Europe, Asia and Australia [10]. WEEV, EEEV and VEEV are zoonotic encephalitic alphaviruses distributed in North and South America and responsible for neurological symptoms in equines and humans [11].

Alphavirus epidemiology is intrinsically related to homophilic arthropod vectors distribution. Among these vectors, mosquitoes of *Aedes* and *Culex* genus are effective contributors of virus to human transmission. In the case of *Aedes*, 2 species *Aedes aegypti* and *Aedes albopictus* are involved. Aedes mosquitoes contribute to *Alphavirus* circulation inside the tropical and subtropical belt but *Aedes albopictus* is prone to geographical expansion to North countries from the late 1970s and spreading from East European countries to Mediterranean countries [12]. This is of great public health concern because of the risk of emergence that rises in novel temperate territories such as metropolitan France [13].

Human *Alphavirus* infections may be related to arthritogenic (Old World) alphaviruses or encephalitic (New World) alphaviruses. Classically, Old World alphaviruses like CHIKV cause an infection including a 4–7 days incubation period followed with the apparition of clinical signs including strong fever, headache, myalgia, arthralgia, maculopapular rash, edemas, abdominal pains and encephalitis in 5% of the [14]. New World alphaviruses like VEEV are responsible for central nervous system (CNS) disorders including severe encephalitis [15, 16]. Moreover, *Alphavirus* infections remain sometimes asymptomatic or can lead to chronic arthritic features over time [17].

Since 2006, imported CHIKV human cases were annually reported in France during the summer with limited autochthonous virus circulation in some cases. This was related to recreational activities and air travels over the world in CHIKV endemic regions [18–20]. This raised a concern about the ability to efficiently detect and report new emergence when it occurred in non-endemic geographical areas.

*Alphavirus* emergence is subjected to many determinants. CHIKV outbreak in Indian Ocean in 2005–2006 provided an example of adaptation of virus to vector. The adaptive CHIKV E1-A226V mutation was responsible for higher infectivity and virus transmission by *Aedes albopictus* [21–24]. The same observation was made with viruses from VEEV antigenic complex. Comparative viral replication studies showed Fort Morgan virus has lost its potential to infect mosquito cells because of two mutations occurring in nsP3 and nsP4, the latter impacting virus replication [25]. Virus adaptation resulted in the acquisition of a new host species through nucleotide changes and reflected the possibility for alphaviruses to jump species barriers and to promote new transmission cycles in case of emergence or resurgence.

Given that human *Alphavirus* infections may result in a non-specific syndrome it is difficult to accurately diagnose these infections. Other arboviruses including *Flavivirus, Bunyavirus, Rhabdovirus* and *Reovirus* can lead to the apparition of the same symptoms in humans and this is also reported for infection by pathogenic leptospira and *Plasmodium*. Detection of virus emergence is sometimes complicated by the cocirculation of 2 distinct arboviruses circulating concomitantly in the same geographical area or by successive outbreaks involving different viruses of *Alphavirus* or *Flavivirus* genus. In French Polynesia, successive outbreaks of DENV, CHIKV, and ZIKA virus were reported in the past five years. Moreover, RRV circulation was serologically reported supporting autochthonous RRV circulation in a silent manner [26].

Differential diagnosis is often necessary to identify the infectious causative agent. Moreover, in case of a new emergence, an extensive panel of pathogen-specific detection tests can be difficult to set up. To overcome this constraint a genus-specific test is required to identify clinical samples positive for alphaviruses. In the acute phase of infection with high levels of viremia, RT-PCR is best suited for virus detection. In the last two decades, several nested or hemi-nested RT-PCR assays were designed to detect members of *Alphavirus* genus [27–30]. For this purpose, nsP1 and nsP4 gene were targeted to design an amplicon. In 2010, Grywna has tested a new primer design based upon nsP4 sequence displaying broader specificity for *Alphavirus* and good clinical sensitivity [31]. Unfortunately, the nested PCR approach used was time-consuming, subjected to risk of cross-contamination and requiring a gel electrophoresis step for amplicon visualization. We herein present a new real time RT-PCR assay based upon hydrolysis of a TaqMan® fluorescently labeled probe that allows rapid and specific detection of genomic *Alphavirus* nsP4 sequences with high sensitivity. Moreover, the format used for the assay design is compatible with routinely implemented molecular tests in clinical laboratories and will simplify diagnosis exclusion.

## Methods

### Ethics approval

Written informed consent was obtained from healthy subjects and patients undergoing arbovirus infection screening and attending Reunion Island University Hospital. The study was approved by the Human Ethics Committee of University of Bordeaux (‘*Comité Consultatif de Protection de Personnes se prêtant à des Recherche Biomédicales’*, Bordeaux France, ref. 2008-A00151–54).

### Reference samples

Virus were obtained from several laboratories. SFV was from Pr Andres Merits in Estonia. ONNV, DENV species 1 to 4 and ZIKA virus were obtained from National Reference Laboratory for Arbovirus of Marseilles (France). RRV and SINV were purchased from the National Collection of Pathogenic Viruses (NCPV, UK). CHIKV strain clone #4.2 was isolated from a clinical sample in our laboratory during the CHIKV outbreak that occurred in Reunion Island in 2005 [32]. Other viruses used for validation studies were isolated from laboratory clinical samples. Rubella virus was detected using Priorix® (GlaxoSmithKline) a trivalent vaccine made of attenuated viral strains for measles virus, *Myxovirus parotidis* and *Rubella.*

Additionally we used RNA standards for quantification of WEEV, EEEV, VEEV, BFV and CHIKV. RNA standards were purchased from Eurogentec and designed with RNA nucleotides using specific viral sequences selected at the location corresponding to the expected amplicon generated by our RT-PCR assay. Accession number of selected sequences was AM258995 (CHIKV), NC001786 (BFV), GQ287640 (WEEV), KC344475 (VEEV), KP282670 (EEEV).

### Nucleic acid isolation

Total nucleic acids were extracted from 200 μL aliquots of plasma human samples or supernatant from cell cultures using Nuclisens® reagents and EasyMAG® nucleic acid isolation platform according to Biomerieux’s recommendations. Final elution was done in 50 μL.

### Design of primers and TaqMan® probes

Alphavirus genomic sequences were available from Virus Pathogen Database and Analysis Resource [33]. Sequence annotation was done using BioEdit Sequence Alignment Editor v7.1.3. Sequence alignment was performed with a Clustal tool provided as a plugin in BioEdit software [34]. Melting temperature and complementarity of primers were checked with Oligocalc calculator [35]. LNA™ probes were designed with LNA™ oligo tools from Exiqon.

To efficiently prime RT-PCR for pan-Alphavirus detection, we applied a degenerate primer design according to Li [36] and consisting of the following steps: 456 genomic downloaded sequences were used to generate consensus sequences specific to each of the 19 Alphavirus species mentioned in Table 1.

**Table 1:** Species-specific consensus sequence determined from 456 viral genomes

Virus	Number of viral genomes used for consensus sequence	Virus	Number of viral genomes used for consensus sequence
CHIKV	188	BFV	4
ONNV	5	BEBV	2
WEEV	12	FMV	3
VEEV	140	HJV	4
EEEV	14	WHAV	2
SINV	23	MIDV	1
RRV	14	NDUV	2
SFV	14	SESV	2
MAYV	3	SPDV	15
GETV	8		

Clustal alignment of available genomic sequences was performed for each *Alphavirus*

A consensus sequence was determined for each viral species using Bioedit software

Consensus sequences were then aligned using CHIKV LR2006 sequence as a ruler guide

**Fig. 1:**
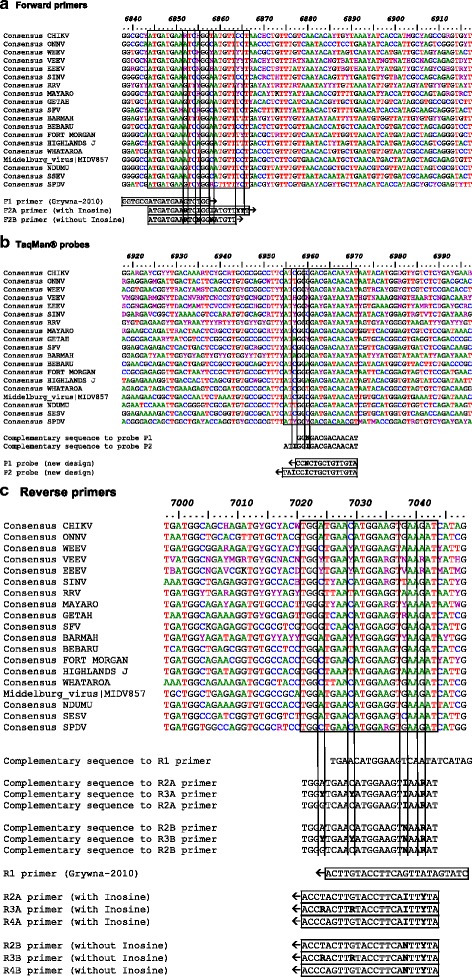
Design of primers and probes in *Alphavirus* nsP4 gene. Genome positions refer to CHIKV LR2006_OPY1|DQ443544 sequence. *Arrows* indicate the 5’➔3′ orientation. Location of forward primers (panel **a**), probes (panel **b**) and reverse primers (panel **c**) used for pan-alphavirus RT-PCR is indicated in *boxes*. *Vertical bars* delineate in primers wobble nucleotides that are considered for degeneracy. Use of inosine containing oligonucleotides was an alternative to decrease primers degeneracy and thus improve sensitivity of detection assay

Alignment of consensus sequences was a prerequisite to select a nucleotide sequence with high similarity between viruses tested in our study. As mentioned by Grywna, nsP4 gene appeared to be highly conserved and thus acted as a good candidate for amplicon design [31]. Using Grywna’s primers as a starting point, we designed our own primers avoiding as much as possible degeneracy (See Table 2 and Fig. 1).

**Table 2:** List of primers and probes

Name	Fluorochrome	Sequence (5’➔ 3′)	Quencher	Nt	Tm (°C)	Degeneracy
F1		GGTGCGATGATGAAGTCTGG		20	60.5	0
R1		CTATGATATTGACTTCCATGTTCA		24	58.3	0
F2A		ATGATGAA**R**TC**I**GG**I**ATGTT**YY**T		23	53.9–59.2	8
F2B		ATGATGAA**R**TC**N**GG**N**ATGTT		20	50.2–56.4	32
R2A		AT**Y**TT**I**ACTTCCATGTTCATCCA		23	55.5–57.6	2
R3A		AT**Y**TT**I**ACTTCCAT**R**TTCA**R**CCA		23	53.9–59.2	8
R4A		AT**Y**TT**I**ACTTCCATGTTGACCCA		23	57.6–59.2	2
R2B		AT**Y**TT**N**ACTTCCATGTTCATCCA		23	55.5–59.2	8
R3B		AT**Y**TT**N**ACTTCCAT**R**TTCA**R**CCA		23	53.9–60.9	32
R4B		AT**Y**TT**N**ACTTCCATGTTGACCCA		23	57.6–60.9	8
P1	ATTO425	AT + GTT + GTC + GT + C**N** + CC	BHQ1/LNA™	14	40.8–43.7/66–69	4
P2	ATTO425	AT + GTT + GTC + GT + C**I**C + C**I**AT	BHQ1/LNA™	17	47.5/67	0

Variable melting temperature was indicated for degenerate primers. Degenerate nucleotides were shown in bold. Y accounted for C/T, R for A/G, N for A or T or C or G. I was used as an alternative for N. ATTO425 labeled probes were quenched using Black Hole Quencher 1 (BHQ1). Locked Nucleic Acid nucleotides (LNA™) were prefixed with a “+” sign and the resulted increase in Tm was indicated following use of Exiqon™ tool for calculation

Degeneracy of a primer is the total number of sequence combinations it contains. For this purpose, inosine was used as an alternative for N nucleotide into forward and reverse primers with ambiguous nucleotides. To take into account nucleotide variation inside the reverse primer, we designed a mix of 3 similar primers harboring different degenerated nucleotide at ambiguous positions. This allowed us to select for primers with 3′ extremity that will match with viral targeted sequences. Primers’ combinations with or without inosine were ultimately compared (See Table 3). These primers delineated a 200 nt long amplicon with a consensus sequence shared by all members of the *Alphavirus* genus. We designed within the amplified region two probes with a length ranging from 14 to 17 nucleotides and harboring LNA™ modifications to fit thermal requirement of a TaqMan® probe.

**Table 3:** List of primers and probe assayed in RT-PCR reactions

RT-PCR MiX	Forward primer at final concentration of:	Reverse primers at final concentration of:	Probe at final concentration of:
#1 primers from Grywna (2010)	F1, 500 nM	R1, 500 nM	P1, 250 nM
#2 primers with deoxy- INOSINE	F2A, 500 nM	R2A, 250 nM +R3A, 250 nM +R4A, 250 nM	P1, 250 nM
#3 primers without deoxy-INOSINE	F2B, 500 nM	R2B, 250 nM +R3B, 250 nM +R4B, 250 nM	P1, 250 nM
#4 primers from Grywna (2010)	F1, 500 nM	R1, 500 nM	P2, 250 nM
#5 primers with deoxy- INOSINE	F2A, 500 nM	R2A, 250 nM +R3A, 250 nM +R4A, 250 nM	P2, 250 nM
#6 primers without deoxy-INOSINE	F2B, 500 nM	R2B, 250 nM +R3B, 250 nM +R4B, 250 nM	P2, 250 nM

Each mix contains the forward primer, up to 3 reverse primers and a probe as mentioned. Combination of 3 reverse primers accounts for nucleotide variation by limiting the use of degenerated primers

### Real time RT-PCR assay

Pan-Alphavirus assay was performed in a total volume of 10 μL including 2.5 μL of template, 2.5 μL of a 4X mix including selected primers and probe according to Table 3, 2.5 μL of molecular grade water and 2.5 μL of 4X ABI TaqMan® Fast Virus 1-Step Master Mix (Applied Biosystems, 850 Lincoln Centre Drive, Foster City CA94404, Cat #4444434). Oligonucleotides were purchased from Eurogentec®. TaqMan® hydrolysis probes were 5′ labeled using ATTO425 fluorochrome and 3′ quenched with BHQ1 (Black hole Quencher-1). Final concentration of primers and probes were respectively set at 500 μM and 250 μM. Cycling conditions were: 45 °C, 5 min, 98 °C, 20 s and 45 cycles comprising 2 steps, 98 °C, 3 s and 58 °C, 45 s with fluorescence reading using CYAN channel for detection of TaqMan® probe hydrolysis. RT-PCR cycling was set on a Roche LC480-II thermal cycler (Roche Applied Science, 68,298 Mannheim Germany, Cat #05015278001).

### Linearity and PCR Efficiency of the pan-Alphavirus TaqMan® assay

RNA standards corresponding to designed amplicons were diluted at the concentration of 1.35 10^7^ copies/μL. A 10-fold dilution series was prepared from RNA standards for WEEV, EEEV, VEEV, BFV and CHIKV. Ct values plotted against log_10_[concentration] were used for linear regression analysis. The coefficient of correlation was used to appreciate the linearity of the assay. The slope of the regression line was used to calculate the PCR efficiency: E = 100 × (10^–1/slope^ – 1).

### Specificity of pan-Alphavirus TaqMan® assay

Specificity of our TaqMan® assay was tested using 9 viruses which are known to belong to *Alphavirus* genus and 15 unrelated viruses from different lineages (Table 4).

**Table 4:** Virus panel tested with the pan*-Alphavirus* RT-PCR assay

Virus	Ct	Virus	Ct
SINV	16.69	ZIKA	nd
RRV	13.22	HIV1	nd
BFV	15.45	HBV	nd
SFV	16.85	HCV	nd
ONN	10.41	EBV	nd
CHIKV	20.96	CMV	nd
WEEV	18.20	BKV	nd
EEEV	19.08	Flu B	nd
VEEV	18.09	Rubella, MeV, Myxovirus	nd
DENV-1	nd		
DENV-2	nd		
DENV-3	nd		
DENV-4	nd		

Specificity of pan-*Alphavirus* assay was tested on a panel of 20 virus comprising 10 *Alphaviruses* and 15 unrelated viruses (nd, not detectable)

### Sensitivity of pan-Alphavirus TaqMan® assay

Clinical sensitivity of our TaqMan® assay was tested by comparing of the Ct obtained from clinical samples positives for CHIKV. We compared the results obtained using a specific-CHIKV RT-PCR assay from Pastorino and our pan-Alphavirus assay [37]. Alternatively, we estimated the limit of detection (LOD) of our assay using a plasmid containing CHIKV amplicon or RNA standards for WEEV, EEEV, VEEV, BFV, and CHIKV.

### Statistical analysis

Tests comparison was assayed for statistical significance using two-tailed Student test with α-risk set at 0.05 using GraphPad Prism v5.0 (GraphPad Software, La Jolla California USA, https://www.graphpad.com/).

## Results

### Pan-Alphavirus real time RT-PCR setup

**Fig. 2:**
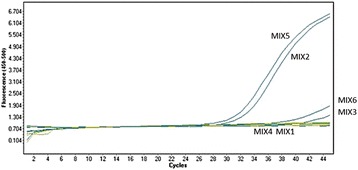
Comparison of 3 primers/probes combinations for pan-*Alphavirus* RT-PCR. Primers containing Inosine were assayed using MIX2 containing probe P1 or MIX5 containing probe P2. Primers without Inosine were tested using MIX3 containing probe P1 or MIX6 containing probe P2. Reference primers from Grywna [31] were used with probe P1 (MIX1) or probe P2 (MIX4)

Nineteen Alphaviruses were used to generate consensus sequences that were aligned prior to primer design in the nsP4 gene. 6 primer combinations were used to check for the effect of the presence of inosine in primers’ sequence. Using CHIKV RNA as a template, we failed to detect nsP4 amplicon with Grywna’s primers with mixes 1, 4 (Fig. 2). This might be due to a potential mismatch at the antepenultimate position in 3′ of the forward primer as evidenced on Fig. 1a. With highly degenerated primers (mixes 3, 6), amplification was weakly detectable (Fig. 2). In contrast, primers enriched with inosine (mixes 2, 5) gave better result as evidenced by earlier Cts and higher fluorescence signals (Fig. 2). From these experiments, we found that the two designed probes worked equally well.

We further investigated the performance of these two probes by calculating ∆Ct_(Mix2-Mix5)_ obtained from 6 CHIKV RNA samples or 6 RRV RNA samples (data not shown). Averaged CHIKV ∆Ct was set at +0.27 and did not account for differences between probes. In contrast, in our pan-Alphavirus assay, averaged RRV ∆Ct at −1.80 clearly indicated a better performance with P1 probe, *ie*, the shorter LNA® probe. Therefore, we decided to select inosine-enriched primers in association with a short LNA® probe and use mix2 for the remaining validation studies of our assay.

### Specificity of Pan-Alphavirus real time RT-PCR assay

**Fig. 3:**
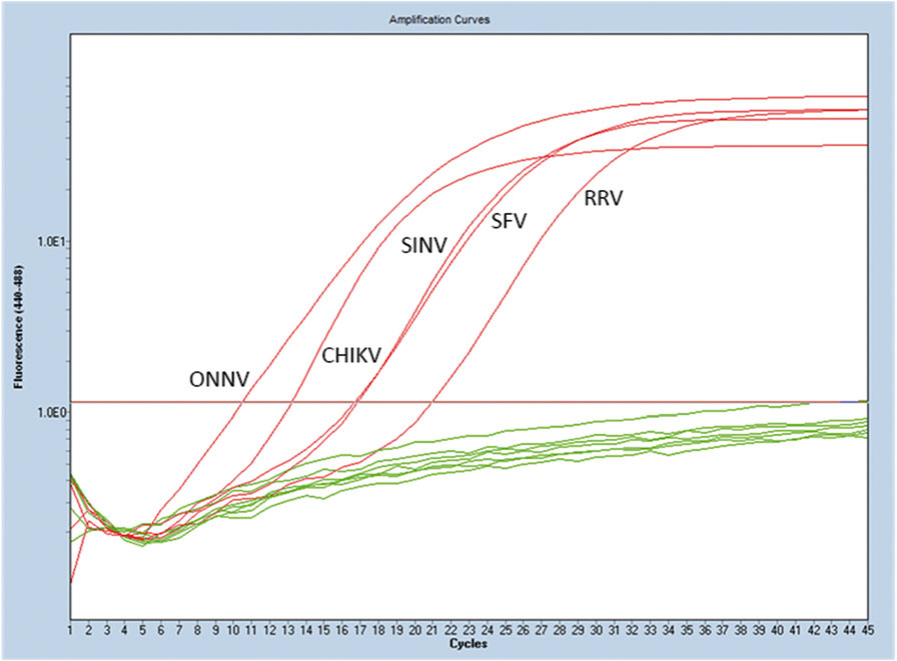
Specific detection of 5 *Alphavirus* species. Positive controls for CHIKV, ONNV, SINV, SFV or RRV were obtained from reference strains or were isolated from clinical samples in our laboratory. Negative controls included water and negative plasma controls. Fluorescence was read on LightCycler 480® (Roche) using Cyan filter (Excitation at 450 nM/Emission at 500 nM)

The specificity for our assay was tested with positive controls for a large panel of viruses including 10 *Alphavirus*. We successfully detected genomic sequence of CHIKV, ONNV, RRV, SINV and SFV (Fig. 3). Unrelated viruses (DENV-1, DENV-2, DENV-3, DENV-4, ZIKA, HIV1, HBV, HCV, EBV, CMV, BKV, Influenza type B, Rubella, MeV, Myxovirus) were not detected with our pan-Alphavirus assay. *Rubella* virus is a *Togavirus* from *Rubivirus* genus. The genus *Rubivirus* is not closely related to the genus *Alphavirus* although they belong to the same family. Using our assay, we were not able to detect *Rubella* virus.

### Sensitivity of Pan-Alphavirus real time RT-PCR assay

**Fig. 4:**
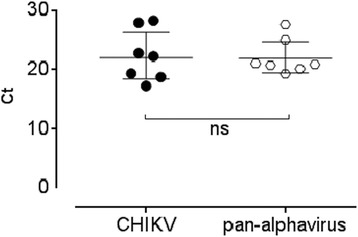
Clinical sensitivity of pan-Alphavirus assay versus CHIKV specific assay. (ns, no statistical difference observed under 5% risk α using a *t*-test)

Clinical sensitivity was assessed based using a set of clinical samples positive for CHIKV that were dually tested using a CHIKV specific TaqMan® assay and our pan-Alphavirus assay. Cts obtained from both assays were similar and no significant difference between the two analyses was detected from data shown in Fig. 4.

The limit of detection of our assay to CHIKV was determined from serial dilutions of a titrated CHIKV RNA. We obtained a LOD at 40 copies per reaction. This was similar to the LOD at 27 copies per reaction reported by Pastorino in his CHIKV TaqMan® assay [37].

In addition we used quantified RNA standards with undergoing serial dilutions prior RT-PCR in order to qualify our assay. (See Table 5).

**Table 5:** Linearity, PCR Efficiency and LOD of the pan-*Alphavirus* RT-PCR assay

	CHIKV	WEEV	EEEV	VEEV	BFV
R^2^	0.994	0.998	0.995	0.993	0.991
Slope	−3.39	−3.81	−3.77	−3.55	−3.67
Intercept	39.49	44.58	46.92	43.35	41.10
Efficiency (%)	97.24	82.99	84.18	91.35	87.12
LOD (copies/run)	26.6	59.5	42.6	111.2	87.5

Linearity of the assay was optimal for each specific RNA standard tested with R^2^ > 0.99. PCR efficiency was under expected range (75% to 110%) and the limit of detection was ranging from 26 to 111 copies/run depending on which RNA standard was used. This was also concordant with the different LODs ranging from 5 to 100 copies per reaction obtained with Grywna’s pan-Alphavirus nested RT-PCR assay [31].

## Discussion

The aim of our study was to implement a new molecular method for *Alphavirus* detection. A couple of RT-PCR methods were already available but were difficult to use in clinical laboratories because of their endpoint PCR format involving amplicon detection. Gel electrophoresis, amplicon sequencing even mass spectrophotometry were used for PCR product visualization. All these methods involved the need to work with open tubes thus raising the risk of cross-over contamination. Our real time RT PCR assay addressed and solved this issue by generating and detecting amplicon at the same time in closed tubes.

Our approach took benefits from careful alignments of available genomic sequences prior to virus-specific consensus sequence generation. In silico analysis was crucial to select for virus consensus sequences, allowing accurate determination of ambiguous nucleotide positions in primers and probes. We especially took into account primers’ nucleotide variation in the 3′ extremity. Primers’ 3′ ends are important for primer extension as unfortunate mismatch in the 5 last 3′ nucleotides usually compromise efficient priming of the PCR reaction. To avoid mismatches between primers and template, 3 strategies were developed: we used degenerated primers; we chose to blast our reverse primer into 3 distinct primers harboring different ambiguous nucleotide combination and to mix them; we used inosine enriched primers instead A or T or C or G containing primers. The latter approach allowed us to select for primers with limited degeneracy [38]. We observed better results with low degenerated primers due to the presence of inosine. This approach may be useful for other pan-PCR system setups.

We also checked for the performance of two Taqman® probe designs incorporating LNA modified nucleotides; the former consisted of a short 14 nucleotide stretch with ambiguous position, the latter was longer with inosine enrichment. The two probes performed equally well but in some cases a better result was obtained with the shorter probe despite of its residual degeneracy. Classically shorter LNA® probes behave better and our design was not affected by a low level of degeneracy.

Our test was validated using clinical samples for CHIKV, ONNV, SINV, SFV and RRV. We also used synthetic RNA standards to detect WEEV, EEEV, VEEV and BFV sequences. Alignments of genomic sequences indicated a potential for the detection of other *Alphaviruses* using this test. These *Alphaviruses* include members of the SFV antigenic complex, *Getah virus* and *Bebaru virus*; members of the VEEV complex, *Fort Morgan virus*, *Highlands J virus*; *Ndumu virus* and *Middelburg virus* that are related to 2 distinct antigenic complexes and responsible for human illness [39]. Interestingly, *Getah virus* infects horses, pigs and goats and Highland J virus is found in horses and poultry [40, 41]. There are some precedents where viruses infecting horses can cross species’ barrier and infect humans. *Hendra virus*, a member of *Henipavirus* genus is not an arbovirus. It is hosted in frugivorous bats causing a zoonosis affecting horses and incidentally humans. Due to increased exposure to natural host’s reservoir, human could contract infection with different and multiple *Alphaviruses* that usually target animals. Risk assessment leading to cross species’ barrier is hard to establish but can result in new virus emergence. The potential of our test to detect unsuspected *Alphavirus* in human related disease may be of great clinical interest.

## Conclusion

TaqMan® assays perfectly suit constraints for virus emergence detection at early stage of disease. In the acute phase of the disease, *Alphavirus* infections are known to cause high viremia in humans and should be easily detectable by RT-PCR. Serological assays could be used in complement at a later stage of the disease [42]. Our test will provide a convenient tool to early incriminate or exclude *Alphavirus* in disease etiology.

## Abbreviations

BEBV: *Bebaru* virus
BFV: *Barmah forest* virus
CHIKV: *Chikungunya* virus
EEEV: *Eastern equine encephalitis* virus
FMV: *Fort Morgan* virus
GETV: *Getah* virus
HJV: *Highlands J* virus
MAYV: *Mayaro* virus
MeV: *Measles* virus
MIDV: *Middelburg* virus
NDUV: *Ndumu* virus
ONNV: *O’nyong nyong* virus
RRV: *Ross river* virus
SESV: *Southern elephant seal* virus
SFV: *Semliki forest* virus
SINV: *Sindbis* virus
SPDV: *Salmon pancreas disease* virus
VEEV: *Venezuelan equine encephalitis* virus
WEEV: *Western equine encephalitis* virus
WHAV: *Whataroa* virus
